# Systemic treatment in advanced soft tissue sarcoma: what is standard, what is new

**DOI:** 10.1186/s12916-017-0872-y

**Published:** 2017-06-02

**Authors:** Anna Maria Frezza, Silvia Stacchiotti, Alessandro Gronchi

**Affiliations:** 1 Department of Cancer Medicine, Fondazione IRCCS Istituto Nazionale dei Tumori, Milan, Italy; 2 Department of Surgery, Fondazione IRCCS Istituto Nazionale dei Tumori, Via Venezian 1, Milan, 20133 Italy

**Keywords:** Sarcoma, Advanced sarcoma, Metastatic sarcoma, Chemotherapy, Tyrosine kinase inhibitors, VEGF inhibitors, Immunotherapy, Survival

## Abstract

For metastatic soft tissue sarcoma (STS) patients not eligible for surgery, systemic treatments, including standard chemotherapy and newer biological compounds, still play the most relevant role in the management of the disease. An anthracycline and alkylating agent combination has formed the cornerstone of chemotherapy in STS for more than 30 years, with its value over that of administration of anthracycline as a single agent still being debated. Efforts have been made to improve the activity and minimise the toxicity of the combination, as well as to explore the upfront efficacy of agents known to be active in sarcoma and to develop new biological compounds. Nevertheless, beyond the first line, evidence for medical treatment in STS is less robust and all the more driven by histology. Thus, the introduction of kinases and small molecule inhibitors in the treatment armamentarium for STS is a major achievement in this setting. Preliminary data on immunotherapy are also available and discussed in this review.

## Background

Soft tissue sarcomas (STS) are a heterogeneous group of rare neoplasms with mesenchymal origin, encompassing approximately 70 different entities [1]. The natural history of these aggressive diseases is characterised by a strong tendency toward local recurrence and metastatic spreading, which occur in 10–30% and 30–40% of patients, respectively, despite optimal initial strategies. The lung is the most common site of STS metastases and pulmonary metastasectomy is the standard treatment for selected patients with limited lung disease. For metastatic patients not eligible for surgery, chemotherapy still plays the most relevant role in the management of the disease. Despite advances over the last decades, the outcome for metastatic patients remains poor, with a median reported overall survival (OS) of 14–17 months [2–4]. In this review, we aim to summarise the results from the most recent studies on metastatic STS and provide indications on the treatment of this rare condition.

## First-line treatment in STS

### Cytotoxic agents

Doxorubicin and ifosfamide have been used in STS for more than 30 years and remain the cornerstone for the treatment of metastatic disease. Nevertheless, whether doxorubicin alone or the combination of doxorubicin and ifosfamide should be used routinely remains debatable. The results from a large, prospective, randomised trial [2], which compared full-dose doxorubicin–ifosfamide versus doxorubicin alone in patients with advanced STS of all types showed no significant difference in OS between groups (14.3 vs. 12.8 months, respectively, *P* = 0.076). Conversely, a significant advantage in both progression free survival (PFS; 7.4 vs. 4.6 months, *P* = 0.003) and overall response rate (ORR; 26% vs. 14%, *P* < 0.0006) was highlighted in the group treated with doxorubicin and ifosfamide. Therefore, it could be reasonable to advocate the use of the combination in fit patients, when disease shrinkage is desirable in order to achieve surgical resection or improve symptom control. Nevertheless, the pooling of several histologies, particularly at a time when different agents have been proven to exert selective activity in specific subtypes, represents a strong limitation in the study [2]. Histology should be considered during decision-making, extending the use of the combination to those subtypes that could benefit more from ifosfamide addition (i.e. synovial sarcoma). Conversely, according to the available retrospective data [5], the activity of ifosfamide is limited in leiomyosarcoma; in this subtype, the combination of doxorubicin and dacarbazine is a potential multi-agent first-line treatment option. Given the key role of doxorubicin in the treatment of STS, several types of anthracycline have been recently tested in first-line treatment, with the view of improving the outcome in patients with advanced disease.

Aldoxorubicin is a novel prodrug of doxorubicin, characterised by a pH-sensitive linker that mediates the binding with endogenous albumin in the bloodstream. The albumin-drug conjugate preferentially localises in the acidic tumour environment where doxorubicin is released, potentially enhancing drug activity and minimising toxicity. In a phase 2b study randomising 123 advanced STS patients to receive doxorubicin or aldoxorubicin [6], the prodrug showed superior efficacy by prolonging PFS (5.6 vs. 2.7 months; *P* = 0.02) and improving ORR (25% vs. 0%). A first-line phase Ib study is currently on-going to evaluate the safety and activity of aldoxorubicin in association with ifosfamide (NCT02235701).

Amrubicin, a third generation anthracycline, has been suggested to be less toxic than doxorubicin, with an ex vivo study proving a lower accumulation in human myocardial strips and a lower tendency to cause cellular oxidative damage [7, 8]. A phase II study with single-agent amrubicin in 24 chemotherapy-naïve STS patients showed a ORR (13%) and a PFS (5.8 months) similar to frontline doxorubicin, with proven tolerability up to a higher cumulative dose [9]. However, a randomised, controlled study assessing non-inferiority of amrubicin compared to the standard is still lacking. Interestingly, a remarkably durable response in a patient with a TLS-CHOP translocated myxoid liposarcoma was noticed [9].

Similarly to that reported with aldoxorubicin and amrubicin alone, attempts to identify newer DNA-alkylators to maximise the efficacy of the combination has not yet led to substantial progress. Palifosfamide, the active metabolite of ifosfamide, does not require prodrug activation and avoids the generation of toxic metabolites. Its efficacy was explored in the PICASSO III study [3], which randomised 447 previously untreated STS patients to receive doxorubicin and palifosfamide or doxorubicin and placebo. Unfortunately, this phase 3 study was unable to confirm the encouraging results of the previous phase 2 trial [10], failing to show any improvement in PFS for the experimental arm (6.0 vs. 5.2 months; *P* = 0.19). Likewise, the phase 3 study from SARC exploring the value of adding evofosfamide, a prodrug preferentially activated under hypoxic conditions, to doxorubicin, did not show any advantage in OS (18.4 vs. 19.0 months; HR = 1.06) and PFS (6.3 vs. 6.0 months; HR = 0.85) for the combination compared to single agent doxorubicin [11].

By adopting the experience developed in refractory STS, the upfront administration of compounds known to be active in further lines has also been explored. Although taxanes are inactive in most subtypes, the combination of gemcitabine and docetaxel (GD) has shown activity in STS, probably due to a synergistic action between the two drugs, and is used in relapsed STS after failure of at least one line of chemotherapy. With the aim of assessing the value of GD as a first-line treatment, a comparative phase 3 study (GEDDIS trial) was run [12], randomising 257 advanced STS patients to receive upfront doxorubicin or GD. The study confirmed the superiority of single agent doxorubicin in terms ORR (65.9% vs. 58.6%) and tolerability, with similar PFS (23 vs. 24 weeks).

Trabectedin, a tetrahydroisoquinoline alkaloid currently approved both in Europe and USA for the treatment of advanced, refractory STS, also failed to show superiority as first-line treatment over doxorubicin in two phase 2 studies [13, 14]. The phase 2b TRUSTS trial, comparing doxorubicin with trabectedin single agent administered over two different schedules (3- and 24-h infusion), was terminated due to lack of superiority in both trabectedin treatment arms as compared to the control arm [13]. Likewise, a phase 2 study from the Spanish Sarcoma Group comparing doxorubicin single agent with the combination of doxorubicin and trabectedin was stopped for futility after the interim analysis (median PFS was 5.5 and 5.7 months in the control and experimental arm, respectively) [14].

Similar results have been reported in a first-line phase 2 study comparing brostallicin, a DNA minor-groove binder, with doxorubicin [15]. Despite being well-tolerated, brostallicin was inferior to doxorubicin both for survival (1-year PFS; 6.5% vs. 15.6%; 1-year OS 50.5% vs. 57.9%) and objective response rate (RR; 3.9% vs. 22.2%).

The results from the studies above underline how, despite efforts over the last years, no regimens have succeeded in providing convincing evidence of superiority as upfront treatment compared to doxorubicin, which remains the standard of care, with or without the association with ifosfamide.

### Monoclonal antibodies

A possible breakthrough in the first-line setting is represented by the recently published results of an open-label phase 1b/2 trial comparing olaratumab and doxorubicin versus doxorubicin alone for treatment of chemotherapy-naïve STS patients [4]. Olaratumab is a recombinant human monoclonal antibody that specifically targets PDGFRα, blocking PDGF-AA, PDGF-BB and PDGF-CC binding and receptor activation. Preclinical data suggest how olaratumab, alone or in combination with doxorubicin, might exert anti-tumour activity in human sarcoma xenograft models [16]. The results of the phase 1b/2 study, randomising 133 patients to receive olaratumab plus doxorubicin or doxorubicin alone, showed a median PFS of 6.6 (95% CI, 4.1–8.3) and 4.1 months (2.8–5.4), a median OS of 26.5 (20.9–31.7) and 14.7 months (9.2–17.1), and an objective RR of 18.2% (9.8–29.6) and 11.9% (5.3–22.2), respectively [4]. The addition of olaratumab to doxorubicin resulted, for the first time, in a clear advantage in OS. The drug has been granted ‘accelerated approval’ status by the Food and Drugs Administration and it has been recommended by the European Medicines Agency for conditional approval. However, the large disparity between OS (11.8 months) and PFS benefit (2.5 months) raised important questions on the drug’s mechanisms of action and the reliability of results. The discrepancy may be partially attributed to differences in treatment duration independent of radiological progression, imbalances in histological subtypes, subsequent therapies, and death due to unrelated events. A confirmatory phase 3 study, the ANNOUNCE (NCT02451943), was run and is fully enrolled, with results awaited in the next months. Additionally, a phase 1b study (NCT02783599) is on-going to evaluate the modulation of biological markers in STS patients receiving olaratumab and doxorubicin, with the aim of gaining a better insight on olaratumab’s mechanism of action.

The prospective evidence for first-line systemic treatment in STS is summarised in Table 1.

**Table 1:** First-line treatment in soft tissue sarcomas: prospective evidence

Study	Study phase	Drug and schedule	Patients	Overall response rate (%)	Progression-free survival (months)	Overall survival (months)
Cytotoxic agents						
Judson et al., 2014 [2]	III	Arm A: D (75 mg/m^2^) 3-weekly Arm B: D (25 mg/m^2^/day, days 1–3) + I (10 g/m^2^ over 4 days) 3-weekly	Arm A: 228 Arm B: 227	Arm A: 14 Arm B: 26	Arm A: 4.6 Arm B: 7.4	Arm A: 12.8 Arm B: 14.3
Chawla et al., 2015 [6]	IIb	Arm A: D (75 mg/m^2^) 3-weekly Arm B: Aldoxorubicin (350 mg/m^2^) 3-weekly	Arm A: 40 Arm B: 83	Arm A: 0 Arm B: 25	Arm A: 2.7 Arm B: 5.6	Arm A: 14.3 Arm B: 15.8
Gupta et al., 2016 [9]	II	Amrubicin 40 mg/m^2^/day, days 1–3, 3 weekly	24	13	5.8	26
Ryan et al., 2016 [3]	III	Arm A: D (75 mg/m^2^) + P (150 mg/m^2^/day, days 1–3) 3-weekly Arm B: D (75 mg/m^2^) + placebo, 3-weekly	Arm A: 227 Arm B: 221	Arm A: 28.3 Arm B: 19.9	Arm A: 6 Arm B: 5.2	Arm A: 15.9 Arm B: 16.9
Tap et al., 2016 [4]	III	Arm A: Evofosfamide (300 mg/m^2^) + D (75 mg/m^2^), 3-weekly Arm B: D (75 mg/m^2^) 3-weekly	Arm A: 317 Arm B: 323	Arm A: 28.4 Arm B: 18.3	Arm A: 6.3 Arm B: 6	Arm A: 18.4 Arm B: 19
Seddon et al., 2015 [12]	III	Arm A: G (625 mg/m^2^ day 1 and 8) + Doc (75 mg/m^2^ day 8), 3-weekly Arm B: D (75 mg/m^2^), 3-weekly	Arm A: 128 Arm B: 129	Arm A: 58.6 Arm B: 65.9	Arm A: 5.6 Arm B: 5.3	Arm A: 14.7 Arm B: 16.5
Bui-Nguyen et al., 2015 [13]	II b	Arm A: T (1.3 mg/m^2^) 3-hour infusion, 3-weekly Arm B: T (1.5 mg/m^2^) 24-hour infusion, 3-weekly Arm C: D (75 mg/m^2^) 3-weekly	Arm A: 47 Arm B: 43 Arm C: 43	Arm A: 14.8 Arm B: 4.7 Arm C: 25.6	Arm A: 2.8 Arm B: 3.1 Arm C: 5.5	NA
Martin-Broto et al., 2016 [14]	II	Arm A: T (1.1 mg/m^2^) 3-hours infusion + D (60 mg/m^2^), 3-weekly Arm B: D (75 mg/m^2^), 3-weekly	Arm A: 54 Arm B: 59	Arm A: 17 Arm B: 17	Arm A: 5.7 Arm B: 5.5	Arm A: 13.3 Arm B: 13.7
Gelderblom et al., 2014 [15]	II	Arm A: Brostallicin (10 mg/m^2^), 3-weekly Arm B: D (75 mg/m^2^), 3-weekly	Arm A: 79 Arm B: 39	Arm A: 3.9 Arm B: 22	Arm A: 1.6 Arm B: 6	NA
Biological agents						
Tap et al., 2016 [11]	Ib/II	Arm A: Olaratumab (15 mg/kg) day 1 and 8 + D (75 mg/m^2^); 3-weekly Arm B: D (75 mg/m^2^), 3-weekly	Arm A: 66 Arm B: 67	Arm A: 18.2 Arm B: 11.9	Arm A: 6.6 Arm B: 4.1	Arm A: 26.5 Arm B: 14.7

*D* doxorubicine; *I* ifosfamide; *P* palifosfamide; *G* gemcitabine; *Doc* docetaxel; *T* trabectedin; *NA* not available

### Tyrosine-kinase inhibitors

Dermatofibrosarcoma protuberans (DFSP) is marked by a translocation resulting in the *COL1A1/PDGFB* fusion gene, responsible for platelet derived growth factor beta-receptor (PDGFRB) activation [17, 18]. This rare STS subtype is characterised by a high tendency toward local aggressiveness and low metastatic potential, which is predominantly associated to the presence of a more aggressive, fibrosarcomatous (FS) component. Imatinib mesylate is highly active in this histology (ORR, 60–70%), it is currently approved and recommended as upfront treatment. FS-DFSP maintains the translocation and is sensitive to imatinib, and should be therefore considered as a first-line option. The RR in patients with FS-DFSP on imatinib is high (approximately 80%), but responses tend to be shorter compared to the classic subtype [19, 20]. Alveolar soft part sarcoma (ASPS) and solitary fibrous tumour (SFT), especially the malignant variant lacking a dedifferentiated component, show limited sensitivity to standard chemotherapy [21, 22]. Angiogenesis has been shown to play a crucial role in the pathogenesis of these subtypes, and encouraging results have been reported with sunitinib and pazopanib in pre-treated patients. Based on the above, there is a rationale to believe that both ASPS and SFT may benefit from the upfront use of antiangiogenic tyrosine kinase inhibitors (TKIs). A prospective phase 2 study exploring pazopanib activity in first-line treatment of SFT is ongoing (NCT02066285).

## Second and further lines in STS

### Cytotoxic agents

The evidence for treatment of metastatic STS after the first line is predominantly built on phase 2 studies suggesting a selective activity of different agents in specific sarcoma subtypes. Gemcitabine is active in refractory STS, more convincing in leiomyosarcoma, angiosarcoma and, to some extent, pleomorphic sarcoma [23]. Conflicting evidence are available on the advantage of a GD regimen over gemcitabine alone, whose better tolerability makes it more appealing in a palliative setting [24, 25]. The activity of gemcitabine in combination with vinorelbine or dacarbazine has also been explored. In a phase II study including adult STS of all types, the combination of gemcitabine and vinorelbine resulted in a clinical benefit rate of 25% [26]; one complete radiological response lasting more than 1 year in a patient with high-grade pleomorphic spindle-cell sarcoma was also reported. In the same population, gemcitabine and dacarbazine compared favourably with dacarbazine single agent in terms of median PFS (4.2 vs. 2 months, *P* = 0.005), OS (16.8 vs. 8.2 months, *P* = 0.014) and clinical benefit rate (49% vs. 25%, *P* = 0.009) [27]. Paclitaxel alone is active in angiosarcoma. Preclinical data suggest that β-blockade induces apoptosis in malignant vascular tumour cells and results in a significant reduction of angiosarcoma growth in in vivo tumour models [28]. Propanolol, alone or in combination with metronomic chemotherapy, has been reported to induce responses in advanced pretreated angiosarcoma patients [29, 30]. Synovial sarcoma is particularly sensitive to ifosfamide [31–33]. As previously mentioned, trabectedin is currently approved in Europe, USA and Japan for the treatment of advanced STS refractory to anthracycline. Leiomyosarcoma, liposarcoma and synovial sarcoma have a higher sensitivity to trabectedin compared to other STS subtypes [34, 35]. Among liposarcomas, an extremely heterogeneous family of STS, myxoid liposarcoma is known to be marked by the t(12;16)(q13;p11), detected in more than 90% of cases. In this subtype, the drug has been proven to exert an additional ‘targeted’ mechanism of action, promoting tumour differentiation through the inactivation of the FUS-CHOP oncogene [36]. This accounts for an activity of the drug in myxoid liposarcoma significantly above the average shown is the other STS subtypes [37]. Similarly to trabectedin, eribulin mesylate is a synthetic analogue of halichondrin B, belonging to the family of microtubule-targeting agents. In addition to its cytotoxic effect, eribulin has been shown to promote vascular remodelling and reversal of the epithelial–mesenchymal transition [38, 39]. A phase 3 trial comparing eribulin with an active control, dacarbazine, in patients with advanced leiomyosarcoma and liposarcoma reported and improvement in OS for patients receiving eribulin (13.5 vs. 11.5 months, respectively; *P* = 0.0169), with the greatest benefit observed in patients with liposarcoma (median OS, 15.6 vs. 8.4) [40]; median PFS was similar in both treatment groups (2.6 months). The positive effect on OS compared with absence of impact on PFS might be partially explained by the biological effects of the drug on vascularisation and microenvironment, which could potentially enhance response to subsequent treatments. In 2016, the Food and Drugs Administration approved eribulin for the treatment of patients with advanced liposarcoma progressing on anthracycline. A phase 3 study evaluating aldoxorubicin compared to investigator’s choice in 433 patients with relapsed or refractory STS (NCT02049905) failed to demonstrate a significant improvement in PFS over the entire study population (full data are expected soon). However, preliminary results suggest a significant prolongation favouring aldoxorubicin for leiomyosarcoma and liposarcoma; final results are awaited in the upcoming months.

Aside from the introduction of new drugs such as eribulin, the tendency toward medical treatment being increasingly driven by histology is considered a major determinant in the OS improvement of advanced STS [41–43]. The selective activity of cytotoxic agents and newer compounds across STS histologies is summarised in Table 2.

**Table 2:** Histology-driven approach in soft tissue sarcomas

Histology	Cytotoxic compounds with selective activity	Target-therapies with selective activities
Leiomyosarcoma	Gemcitabine ± docetaxel, trabectedin, dacarbazine	Pazopanib
Dedifferentiated liposarcoma	High-dose ifosfamide, trabectedin, eribulin	
Myxoid liposarcoma	Trabectedin, eribulin	
Synovial sarcoma	Ifosfamide, trabectedin	Pazopanib
Epithelioid sarcoma	Gemcitabine	Pazopanib
Angiosarcoma/intimal sarcoma	Gemcitabine, paclitaxel	Pazopanib, sorafenib
Alveolar soft part sarcoma		Pazopanib, sunitinib, cedinarib
Solitary fibrous tumour	Dacarbazine	Pazopanib, sunitinib
Clear cell sarcoma		Pazopanib, sunitinib
Extraskeletal myxoid chondrosarcoma		Pazopanib, sunitinib
Perivascular epithelioid cell tumor	Gemcitabine	m-TOR inhibitors
Epithelioid hemangioendothelioma		Pazopanib, m-TOR inhibitors, interferon
Inflammatory myofibroblastic tumour		Crizotinib
Undifferentiated pleomorphic sarcoma	High-dose ifosfamide, gemcitabine	
Dermatofibrosarcoma protuberans		Imatinib, sorafenib, sunitinib

### Kinase inhibitors

One of the most encouraging fields of development in STS over the last years has been that of the introduction of kinase-inhibitors in the treatment armamentarium, as exemplified by imatinib for the treatment of gastrointestinal stromal tumours [44]. Prospective evidence for kinase inhibitors in STS is summarised in Table 3.

**Table 3:** Kinase-inhibitors in soft tissue sarcomas: prospective evidence

Study	Population	Study phase	Drug and schedule	Patients	Overall response rate (%)	Progression-free survival (months)	Overall survival (months)
Tyrosine-kinase inhibitors							
George et al., 2009 [60]	Metastatic all-type STS	Phase II	Sunitinib 37.5 mg once daily	53	2	1.8	NA
Hensley et al., 2009 [59]	Advanced uterine LMS	Phase II	Sunitinib 50 mg daily, 4 weeks on, 2 weeks off	25	9	1.5	NA
Rutkowski et al., 2010 [19]	DFSP	Pooled analysis of two phase II	Imatinib 400–800 mg daily	34	32	20.4	Unreached
Wagner et al., 2012 [68]	MTF-associated tumours (including ASPS, CCS)	Phase II	Tivantinib 120 mg, twice daily	47	2	3.6	NA
van der Graaf et al., 2012 [45]	Metastatic, non-adipocytic STS	Randomised, phase III	Arm A: pazopanib 800 mg daily Arm B: placebo	Arm A: 246 Arm B: 123	Arm A: 6 Arm B: 0	Arm A: 4.6 Arm B: 1.6	Arm A: 12.5 Arm B: 10.7
Ray-Coquard et al., 2012 [55]	Advanced superficial (stratum A) and visceral (stratum B) angiosarcoma	Phase II	Sorafenib 400 mg twice daily	Stratum A: 26 Stratum B: 15	Stratum A: 15 Stratum B: 13	Stratum A: 1.8 Stratum B: 3.8	Stratum A: 12 Stratum B: 9
Kummar et al., 2013 [61]	Advanced ASPS	Phase II	Cedinarib 30 mg daily	46	35		
Mir et al., 2016 [54]	Cohort A: LPS Cohort B: LMS Cohort C: SS Cohort D: other sarcomas	Randomised, phase II	Arm A: regorafenib 160 mg/day 3 weeks on, 1 week off Arm B: placebo	Cohort A: 43 Cohort B: 56 Cohort C: 27 Cohort D: 56	Cohort A: 0 (arm A) vs. 0 (arm B) Cohort B: 0 (arm A) vs. 4 (arm B) Cohort C: 8 (arm A) vs. 0 (arm B) Cohort D: 11 (arm A) vs. 0 (arm B)	Cohort A: 1.1 (arm A) vs. 1.7 (arm B) Cohort B: 3.7 (arm A) vs. 1.8 (arm B) Cohort C: 5.6 (arm A) vs. 1 (arm B) Cohort D: 2.9 (arm A) vs. 1 (arm B)	Cohort A: 4.7 (arm A) vs. 8.8 (arm B) Cohort B: 21 (arm A) vs. 9.1 (arm B) Cohort C: 13.4 (arm A) vs. 6.7 (arm B) Cohort D: 12.1 (arm A) vs. 9.5 (arm B)
Chi et al., 2016 [62]	Metastatic all-type STS	Phase II	Anlotinib 12 mg daily, 2 weeks on, 1 week off	166	11	5.6	NA
Agulnik et al., 2016 [87]	Metastatic all-type STS	Phase II	Tivozanib 1.5 mg daily, 3 week on, 1 week off	58	3.6	3.5	12.2
Schuetze et al., 2016 [67]	Metastatic all-type STS	Phase II	Dasatinib 100 mg twice daily	200	1	1.9	8
Schuetze et al., 2017 [66]	Metastatic ASPS, CS, chordoma, ES, SFT	Phase II	Dasatinib 100 mg twice daily	116	<1	5.8	21.6
Serine/threonine kinase inhibitors							
Demetri et al., 2013 [69]	Metastatic all-type STS and BS (responsive/stable on CHT)	Randomised, phase III	Arm A: ridaforolimus 40 mg once daily, 5 days every week Arm B: placebo	Arm A: 347 Arm B: 364	NA	Arm A: 17.7 Arm B: 14.6	Arm A: 21 Arm B: 19.8
Schwartz et al., 2013 [76]	Metastatic all-type STS and BS	Phase II	Cixutumumab (6 mg/kg) and temsirolimus (25 mg)	174	5	NA	NA
Dickson et al., 2013 [80]	Advanced CDK4-amplified WDLPS/DDLPS	Phase II	PD0332991 200 mg orally once daily, 2 weeks on, 1 week off	30	3	18	NA

*ASPS* alveolar soft part sarcoma; *BS* bone sarcomas; *CCS* clear cell sarcoma; *CHT* chemotherapy; *CS* chondrosarcoma; *DDLPS* dedifferentiated liposarcoma; *DFSP* dermatofibrosarcoma protuberans; *ES* epithelioid sarcoma; *LMS* leiomyosarcoma; *LPS* liposarcoma; *MTF* microphthalmia transcription factor; *NA* not available; *OS* osteosarcoma; *SFT* solitary fibrous tumour; *SS* synovial sarcoma; *STS* soft tissue sarcomas; *UPS* undifferentiated pleomorphic sarcoma; *WDLPS* well-differentiated liposarcoma

#### Tyrosine kinase inhibitors (TKIs) targeting angiogenesis

A variety of TKIs exert their antitumor effect by targeting angiogenesis. Pazopanib, a TKI targeting VEGFR 1–3, PDGFRA, PDGFRB and KIT, was tested in advanced, pre-treated STS patients, and showed an improvement in PFS of 3 months compared to placebo [45]; a good performance status and a low or intermediate tumour grade were selected as favourable prognostic factors. Liposarcomas were excluded from the study based on the negative results reported in a previous phase 2 study for this histology [46]. The results of the PALETTE study [45] led to pazopanib approval in advanced, refractory non-lipomatous sarcoma. Although the mechanism of action is still poorly understood, pazopanib seems to be more active in leiomyosarcoma, synovial sarcoma, vascular sarcomas (epithelioid hemangioendothelioma and intimal sarcoma), ASPS and SFT [45, 47–49]. Further studies are ongoing to better exploit its activity across STS histologies and evaluate the combination of pazopanib with cytotoxic (i.e. gemcitabine, taxanes) and newer (i.e. anti-endoglin, m-TOR inhibitors) agents [50–53]. Regorafenib, a TKI targeting VEGFR1-3, RET, KIT, PDGFR and Raf, was found to be associated with a minor PFS advantage in non-adipocytic STS progressing on anthracycline in a single phase II study [54].

In addition to pazopanib and regorafenib, several other TKIs targeting angiogenesis have been tested in sarcoma, showing a different activity across histologies. Angiosarcoma and SFT seem sensitive to sorafenib [55]. The anti-tumour activity of sunitinib was shown in ASPS, SFT, clear cell sarcoma and extraskeletal myxoid chondrosarcoma [48, 56–60]; no signs of activity were found in most of the remaining STS subtypes. Encouraging results were reported with cediranib, a potent inhibitor of VEGFR1, VEGFR3 and KIT, in ASPS [61]; in the same subtype, limited evidence is also available for anlotinib [62]. Tivozanib, a TKI targeting VEGFR1-3, PDGFRα/β and cKIT, showed signs of activity in a phase 2 study including 58 heavily pre-treated STS patients (47% leiomyosarcoma) [63]. Responses to sorafenib and sunitinib have been reported in advanced DFSP progressing on imatinib, and a phase 2 study with pazopanib has been recently completed (NCT01059656) [64, 65].

#### Other TKIs

The intracellular tyrosine kinase c-SRC pathway, including as downstream targets EGFR, PDGFR and c-KIT, has been reported to be up-regulated in STS, especially leiomyosarcoma and synovial sarcoma. Despite encouraging preclinical data, negative results have been reported in two phase 2 studies exploring the activity of dasatinib, a potent small molecule inhibitor of SRC, in advanced, pre-treated STS patients [66, 67]. Additionally, the hepatocyte growth factor receptor (MET) and anaplastic lymphoma kinase (ALK) are TK-receptors, whose disruption promotes cellular proliferation, angiogenesis and disease spreading in many solid cancers, including STS. Crizotinib, a TKI targeting both ALK and MET, has shown activity in ALK-rearranged inflammatory miofibroblastic tumours (IMTs), which account for approximately 50% of all IMT cases. The EORTC phase 2 study, CREATE, is currently exploring crizotinib activity in IMT, alveolar rhabdomyosarcoma, clear cell sarcoma and ASPS (NCT01524926). Tivantinib, a selective MET inhibitor, was also tested in a phase 2 study including ASPS and clear cell sarcoma, but only a modest activity was reported [68].

#### Serine/threonine kinase inhibitors

The better comprehension of STS biology led to the identification of several biological mechanisms potentially applicable in new drug development. The value of mammalian target of rapamycin (mTOR) inhibition in STS has been extensively exploited. With the view of prolonging the duration of disease control in patients who achieved a previous stabilisation or response to chemotherapy, the phase 3 SUCCEED trial tested the possible role of ridaforolimus, a serine/threonine kinase inhibitor targeting mTOR, as a maintenance therapy [69]. The study showed only a modest benefit in PFS for the drug compared to placebo (17.7 vs. 14.6 weeks, *P* = 0.001), according to which this approach cannot be recommended in this setting. Across histologies, mTOR-inhibition is known to be active in PEComas, often harbouring genetic aberrations and activation of the TSC1/2–mTOR signalling pathway. The use of sirolimus and temsirolimus in this subtype induces consistent but often short-lasting responses [70–72]. Sirolimus has shown activity in epithelioid hemangioendothelioma, with a reported clinical benefit rate of 56% [73]. Conflicting results have been described for STS of all types on the association between mTOR and IGF1R inhibitors, with more convincing evidence for Ewing sarcoma [74–76]. A possible role for mTOR inhibition in neurofibromatosis type 1-related malignant peripheral nerve sheath tumour (MPNST) has also been postulated [77]. The inhibition of MAPK signalling through salumetinib, an oral selective inhibitor of MAPK kinase 1 and 2, has led to encouraging results in paediatric neurofibromatosis type 1 patients with inoperable plexiform neurofibromas, providing a rationale for its investigation in MPNST [78]. Among serine/threonine kinases, the role of CDK4 has been studied in STS, highlighting an overexpression of the protein in more than 90% of well-differentiated/dedifferentiated liposarcoma (WDLS/DDLS) [79]. The phosphorylation of the retinoblastoma protein allowed by cyclin D/CDK4/6 causes the detachment of retinoblastoma protein from the E2F transcription factor, leading to the transcription of multiple target genes including *MDM2*. The activity of palbociclib, a CDK4/CDK6-inhibitor currently approved in breast cancer, in CDK4-positive WDLS/DDLS was shown in a phase 2 study reporting a 12-week PFS rate of 66% [80]. A second phase 2 study (NCT01209598) testing different doses and schedules to minimise haematological toxicity has recently completed accrual and results are awaited.

### Small molecule inhibitors

As for other small molecule inhibitors, the role of MDM2-antagonists and histone deacetylase inhibitors is currently under investigation. In a proof-of-mechanism study from a French group [81], 20 patients with chemotherapy-naïve primary or relapsed WDLS/DDLS, MDM2 amplified and eligible for resection, received RG7112, a MDM2-antagonist, with one response and 14 stable disease cases being reported, albeit with a significant gastrointestinal and bone-marrow toxicity. A phase 1b/2 study (NCT01605526) is currently evaluating the tolerability and activity of a potentially less toxic compound, RO5045337, in association with doxorubicin. An intriguing new therapeutic approach that is currently under development in STS is represented by the inhibition of histone methylation, resulting in chromatin remodelling and modulation of the resultant transcriptional output. Tazemetostat is a small molecule inhibitor of the histone-lysine methyltransferase EZH2, whose activity is enhanced in integrase interactor 1 (INI1)-deficient tumours [82]. Among STS, genetic loss of INI1 has been reported in epithelioid MPNST, extraskeletal myxoid chondrosarcoma, myoepithelial carcinoma and up to 90% of epithelioid sarcoma [83–85]. INI1 can be deficient in synovial sarcoma (SS) marked by the fusion genes *SS18-SSX1*. The resulting fusion protein causes the displacement of wild-type SS18 and INI1 from the SWI/SNF complex, leading to INI1 proteolytic degradation [86]. Given the preliminary results of the phase 1/2 study showing activity of tazemetostat in INI1 deficient tumours (NCT02601950) [87], a phase 2 trial (NCT02601950) is currently ongoing to assess the activity of the drug in this group of solid cancers, including INI1-deficient epithelioid sarcoma and SS marked by the *SS18-SSX1* translocation.

### Immunotherapy

Immunotherapy has been one of the major breakthroughs in oncology, for both solid and haematological tumours. Despite historical evidence supporting its role in STS, the results currently reported with adoptive immunotherapy and immune synapse blockade remain controversial. NY-ESO-1, a member of the cancer testis family of tumour antigens, is expressed in approximately 80% of SS cases. A multi-cohort pilot study (NCT01343043) is currently testing the activity of genetically engineered NY-ESO-1c259 SPEAR T-cells in HLA-A*02 patients with SS undergoing different lymphodepleting regimens. The preliminary results show a reasonable tolerability and support the activity of the approach in this histology, with responses described independently from the level of NY-ESO-1 expression. The absence of objective responses in the cohort not receiving fludarabine within the preparative regimen let the authors postulate a role for the drug in the induction of response [88]. The activity of pembrolizumab and nivolumab, 2 humanised monoclonal IgG4 antibodies directed against the cell surface receptor PD-1, has also been preliminarily explored, both alone or in combination with cytotoxic and antiangiogenic drugs. In the SARC028 phase 2 study [89], pembrolizumab as a single agents showed activity in unselected STS of all types, with an ORR of 17.5% and a 55% 3-month PFS; undifferentiated pleomorphic sarcoma and DDLS were the histologies that seemed to benefit the most. In the French experience with the same compound [90], one response was reported in a SFT, treated in combination with cyclophosphamide. In a phase 2 study evaluating the activity of nivolumab alone or in association with pazopanib [91], one response in an epithelioid sarcoma patient was recorded in the group treated with the combination, but no signs of activity in STS for the drug as a single agent were reported. Despite an anecdotal response reported, nivolumab as a single agent failed to demonstrate antitumor activity in a phase 2 study on 12 patients with advanced uterine leiomyosarcoma [92, 93]. Nevertheless, the value of immunotherapy in STS is still largely unexplored. Further research is on-going to allow better patient selection and to investigate new combinatorial strategies. Results from the available studies on immunotherapy in STS are summarised in Table 4.

**Table 4:** Immunotherapy in soft tissue sarcomas

Study	Population	Study phase, status	Drug and schedule	Patients	Overall response rate (%)
Mackall et al., 2016 [88]	Synovial sarcoma	I/II, recruiting	NY-ESO-1c259 SPEAR T-cells Cohort 1 and 2: FL 30 mg/m^2^/day, day 1–4; CTX 1800 mg/m^2^/day day 1–2 Cohort 3: CTX 1800 mg/m^2^/day day 1–2 Cohort 4: FL 30 mg/m^2^/day, day 1–3; CTX 600 mg/m^2^/day; day 1–3	Cohort 1: 15 Cohort 2: 2 Cohort 3: 2 Cohort 4: 0	Cohort 1: 50 Cohort 2: NA Cohort 3: NA Cohort 4: NA
Italiano et al., 2016 [90]	LMS (Arm A), UPS (Arm B), GIST (Arm C), OS (Arm D), other sarcomas (Arm E)	II, recruiting in arm B and D	Pembrolizumab 200 mg i.v. 3-weekly; CTX 50 mg BID 1week on, 1 week off	Arm A: 15 Arm B: 0 Arm C: 10 Arm D: 0 Arm E: 16	No objective responses
Burgess et al., 2016 [89]	All-type STS (arm A) and BS (arm B)	II, completed	Pembrolizumab, 200mg i.v., 3-weekly	Arm A: 40 Arm B: 40	Arm A: 17.5 (UPS, LPS, SS) Arm B: 5 (OS, CS)
Paoluzzi et al., 2016 [91]	All-type STS and BS	Retrospective	Arm A: nivolumab 3 mg/kg i.v., 2-weekly Arm B: nivolumab 3 mg/kg i.v., 2-weekly + pazopanib 800 mg/day	Arm A: 10 Arm B: 18	Arm A: 10 (CS) Arm B: 11 (ES, OS)
George et al., 2016 [90]	Leiomyosarcoma	II	Nivolumab 3 mg/kg i.v., 2-weekly	12	No objective responses

*BS* bone sarcomas; *CS* chondrosarcoma; *CTX* cyclophosphamide; *ES* epithelioid sarcoma; *FL* fludarabine; *GIST* Gastrointestinal stromal tumors; *LMS* leiomyosarcoma; *LPS* liposarcoma; *NA* not available; *OS* osteosarcoma; *SS* synovial sarcoma; *STS* soft tissue sarcomas; *UPS* undifferentiated pleomorphic sarcoma

## Conclusions

Doxorubicin remains to date the standard in the treatment of advanced STS. The combination with ifosfamide should be considered upfront in fit patients who might benefit from tumour response and in histologies with selective sensitivity to alkylating agents. Despite encouraging preliminary data, results from the recently completed phase 3 study on olaratumab and doxorubicin are required to confirm the value of this combination in first-line treatment. Beyond the first line, the treatment for STS is being increasingly driven by histology. Newer strategies, including drugs targeting epigenetic mechanisms and immunotherapies, are currently being developed to improve the outcome in this population.
